# A cross-sectional survey on patient safety culture among healthcare providers in the Upper East region of Ghana

**DOI:** 10.1371/journal.pone.0221208

**Published:** 2019-08-20

**Authors:** Alexander Akologo, Aaron Asibi Abuosi, Emmanuel Anongeba Anaba

**Affiliations:** Department of Public Administration and Health Services Management, University of Ghana Business School, Legon, Accra, Ghana; Medical University Graz, AUSTRIA

## Abstract

**Introduction:**

Adverse events pose a serious threat to quality patient care. Promoting a culture of safety is essential for reducing adverse events. This study aims to assess healthcare providers’ perceptions of patient safety culture in three selected hospitals in the Upper East region of Ghana.

**Methods:**

The English version of the Hospital Survey on Patient Safety Culture (HSOPSC) questionnaire was administered to 406 clinical staff. Statistical Package for Social Science (SPSS) software, version 23, was used to analyze the data. The results were presented using descriptive statistics, Pearson Correlation Analysis and One-way Analysis of Variance (ANOVA).

**Results:**

It was found that two out of twelve patient safety culture dimensions recorded high positive response rates (≥ 70%). These include teamwork within units (81.5%) and organizational learning (73.1%). Three patient safety culture dimensions (i.e. staffing, non-punitive response to error and frequency of events reported) recorded low positive response rates (≤ 50%). The overall perception of patient safety correlated significantly with all patient safety culture dimensions, except staffing. There was no statistically significant difference in the overall perception of patient safety among the three hospitals.

**Conclusion:**

Generally, healthcare providers in this study perceived patient safety culture in their units as quite good. Some of the respondents perceived punitive response to errors. Going forward, healthcare policy-makers and managers should make patient safety culture a top priority. The managers should consider creating a ‘blame-free’ environment to promote adverse event reporting in the hospitals.

## Background

Adverse events are ‘unintended injuries or complications that are caused by health care management rather than by the patient’s underlying disease and can lead to death, disability at the time of discharge and prolonged hospital stay’[1, 2]. At least, one adverse event is recorded among every 300 patients worldwide [3]. Adverse events account for more deaths than motor-accident, breast cancer and Acquired Immune Deficiency Syndrome (AIDS) [4]. In this regard, the World Health Organization is urging countries to pay the closest possible attention to the problem of patient safety and to establish and strengthen science-based systems necessary for improving patient safety and quality of care [5, 6].

Studies have shown that promoting patient safety culture among healthcare providers is a key to reducing adverse events and maintaining quality of care [7, 8]. Patient Safety Culture (PSC) is the ‘set of shared values, attitudes, perceptions, beliefs and behaviors that support safe practices among individuals in healthcare organizations’[6]. Healthcare facilities with a positive patient safety culture are characterized by communication founded on mutual trust, shared perceptions of the importance of safety and confidence in the efficacy of preventive measures [7]. According to Richardson and Storr (8), clinicians are among the main drivers of patient safety, because they have direct contact with patients [9, 10].

A positive PSC depends on effective communication, appropriate staffing, procedure compliance, leadership support, non-punitive response to error and teamwork [11]. A positive PSC promotes collaborative learning, reporting of adverse events and ‘blame free’ culture [12]. In addition, healthcare providers should be able to identify and report adverse events without fear of blame [13]. Studies have found that a positive PSC can help reduce adverse events, improve quality of care and increase patients’ and providers’ satisfaction with care [13, 14]. A positive patient safety culture can help reduce unnecessary hospital admissions due to adverse events [15].

Even though adverse events are prevalent in resource-constrained regions in sub-Saharan Africa [16, 17], there is limited knowledge of patient safety culture among healthcare providers in these regions [18]. In Ghana, patient safety culture is an emerging area [17]. Even though efforts are made to prevent adverse events at various levels of care, there is evidence that adverse events occur in Ghana’s health facilities [19, 20]. These include patient falls, injection abscesses, surgical wound infections, hospital-acquired infection/sepsis, hospital-incurred patient accident or injury, unplanned return or visit to the operating theatre during admission, unplanned open surgery following closed or laparoscopic surgery, unexpected death (i.e. not an expected outcome of the disease during hospitalization), and any other undesirable outcomes. Adverse events occur at various levels of care, whether primary, secondary or tertiary level [21, 22]. Adverse events also occur regardless of the ownership of the hospital, whether government, quasi-government, faith-based or private-for-profit [23, 24].

However, there is a dearth of literature on patient safety culture in Ghana. Previous studies on patient safety focused on adverse drug reaction reporting [25–27], improving the accuracy of malaria-related laboratory tests [28], situational analysis of patient safety [20], and patient safety systems and structures [29, 30] with less focus on attitudes, beliefs and behaviors that support safe practices in health facilities. To the best of the authors’ knowledge, no study has been conducted in Ghana using a comprehensive patient safety culture tool such as the Agency for Healthcare Research and Quality hospital-based patient safety culture survey tool-HSOPSC [7].

It is against this background that a comparative study of three hospitals was done in the Upper East region of Ghana. Findings from such a study will enable healthcare providers and managers to gain insight into the nuances of patient safety and learn some best practices in other hospitals. The study sought to find answers to the following research questions: (i) how do healthcare providers in the Upper East region perceive patient safety culture? (ii) are there any significant differences in healthcare providers’ perceptions of patient safety culture in public, private (for-profit) and faith-based (not-for-profit) hospitals?

## Methods

### Study design and setting

A cross-sectional survey was conducted among three hospitals in the Bawku enclave in the Upper East region of Ghana [31]. The Bawku enclave has three administrative districts: Bawku West, Bawku East, and Garu-Temapane. According to the 2010 Population and Housing Census, Bawku has a total population of 322,575, representing 19.6 percent of the total population (1,046,545) of the Upper East region [32]. There are three hospitals in Bawku: a faith-based hospital, a public hospital and a private hospital. Moreover, there are health centers, clinics and Community Health Implementation Programme (CHIP) compounds that complement the three hospitals in providing health care to the populace. The faith-based hospital has a bed capacity of 388 and 304 clinical staff. The private hospital has a bed capacity of 80 and 97 clinical staff and the public hospital has a bed capacity of 110 and 224 clinical staff.

### Population and sampling

The target population for this study was all clinical staff (healthcare providers who have direct contact with patients). The three hospitals had a total of 625 clinical staff. Because of resource-constraints, multistage sampling technique was adopted. Purposive sampling technique was adopted to select three hospitals in Bawku. Convenience sampling technique was adopted to recruit respondents into the study. The purpose of the study was made known to potential respondents and those who were interested and available were selected. This technique seemed more appropriate because of the shift system operated by the hospitals. Respondents comprised clinical staff (nurses, physicians, laboratory technicians, pharmacists among others). The sample size was 239. As a way of increasing the power of the study to detect significant differences, and also cater for non-respondents, 70% (167) of the sample size was added, summing up to 406. Based on the staff strength of the hospitals, a ratio of 3:2:1 was adopted to recruit respondents.

### Inclusion and exclusion criteria

This study included only hospitals and excluded health centers, clinics and health post. Only clinical staff (healthcare providers who have direct contact with patients such as nurses, physicians, pharmacists and laboratory technicians) were included, but excluded healthcare providers who do not have direct contact with patients, such as accountants, drivers, etc.

### Instrument

The English version of Hospital Survey on Patient Safety Culture (HSOPSC) questionnaire, developed by the Agency for Healthcare Research and Quality (AHRQ) was adopted for the study. The HSOPSC questionnaire has 42 items grouped into 12 dimensions. The dimensions include teamwork within units (4 items, i.e. staff work together as a team); supervisors’ expectations and actions promoting patient safety (4 items, i.e. supervisors do not overlook patient safety problems); organizational learning continuous improvement (3 items, i.e. mistakes have led to positive changes); and management support for patient safety (3 items, i.e. hospital management show that patient safety is a top priority). The remaining dimensions are teamwork across unit (4 items, i.e. hospital units coordinate with one another to provide the best care for patients); staffing (4 items, i.e. there are enough staff to handle the workload); handoffs and transitions (4 items, i.e. important patient care information is transferred during shift changes); nonpunitive response to errors (3 items, i.e. staff feel that event reports are not held against them); and overall perception of patient safety (4 items, i.e. procedures and systems are good at preventing errors). Items under these composites are on a five-point Likert scale (1 = Strongly disagree, 2 = Disagree, 3 = Neither, 4 = Agree, 5 = Strongly agree).

Other composite measures are feedback and communication about error (3 items, i.e. staff are given feedback about changes implemented); communication openness (3 items, i.e. staff freely speak up if they see something that may negatively affect a patient); and frequency of events reported (3 items, i.e. mistakes are detected and corrected before affecting patients). Items under these composites are on a five-point Likert scale (1 = Never, 2 = Rarely, 3 = Sometimes, 4 = Most of the time, 5 = Always).

In addition, the HSOPSC questionnaire includes an item that requires respondents to rate their overall perception of patient safety (patient safety grade) on a five-point Likert scale (A = excellent, B = very good, C = acceptable, D = poor, E = failing). Also, respondents are required to state the number of adverse events they have reported over the past 12 months. Demographic information (gender, age, position, work experience, hospital type, etc.) is the last section of the questionnaire. The HSOPSC questionnaire has a Cronbach alpha ranging from 0.63 to 0.84 [33]. To the best of the authors’ knowledge, the questionnaire has not been used in Ghana but has been used in similar contexts such as Nigeria and Ethiopia [34, 35]. The questionnaire was pilot-tested with twelve healthcare providers in a different hospital. The questionnaire was administered to 406 clinical staff on duty by hand and a period agreed upon for pick-up (three to four days). This approach seemed more appropriate because of the busy schedule of the respondents. Data collection took place in February and March 2017.

### Ethical consideration

The study received approval by the University of Ghana College of Humanities Ethics Committee **(ECH 085/17-18).** Permission was sought from the management of the hospitals and verbal or written consent obtained from respondents. Healthcare providers who agreed to participate in the study were asked to sign a consent form. For participants who were not comfortable signing the consent form, verbal consent was accepted. Both the verbal and written consent received the approval of the Ethics Committee. Participation was voluntary and participants had the free will to opt out by not returning the questionnaire.

### Data analysis

Four hundred and six questionnaires were distributed in this survey, of which 384 questionnaires were retrieved, representing 94.5% response rate. The retrieved questionnaires were cross-checked for errors and completeness. The questionnaire was coded into Statistical Package for Social Science (SPSS) software, version 23. The data were cross-checked for wrong and omitted entries by running descriptive for all the variables. Using the Shapiro-Wilk test, the data was found to be normally distributed. Percentages of positive response rates were calculated by adding frequency proportions for strongly agree and agree (for non-negatively worded questions) and strongly disagree and disagree (for negatively worded questions). The composite index for each dimension was the average of positive response rates. Considering that patient safety culture in an emerging area in Ghana, a composite index ≥ 70% was considered a high positive response rate, 69% -51% was considered a moderately positive response rate and ≤ 50% was considered a low positive response rate. Pearson Correlation Analysis was used to explore associations between patient safety culture dimensions (independents variables) and patient safety grade (dependent variable). A composite index was calculated for each dimension by computing the items under them. Prior to that, all negatively worded items such as ‘we have patient safety problems in this unit’, were reversed (5 = strongly disagree, 4 = disagree, 3 = neutral, 2 = agree, 1 = strongly agree). It was expected that majority of the respondents would respond ‘strongly disagree’ or ‘disagree’ to negatively worded items. In addition, One-Way Analysis of Variance (ANOVA) was adopted to compare the mean scores of the three hospitals [36, 37]. All assumptions underlying ANOVA were satisfied.

## Results

### Descriptive statistics

#### Socio-demographic characteristics of respondents

Fifty-six percent of the respondents were females and 73% were diploma holders. More than half (53%) of the respondents were young adults, 82% had less than six years of working experience and 51% of the respondents were nurses. Half of the respondents worked between 40–50 hours per week. Details are shown in Table 1.

**Table 1:** Demographic characteristics of 384 clinical staff in the Upper East region of Ghana, 2017.

Demographic characteristics		Respondents (n = 384)	Frequency (%)
Gender			
	Male	167	44
	Female	217	56
Education			
	Certificate	38	10
	Diploma	280	73
	Degree	66	17
Age			
	18–30 years	202	53
	31–40 years	136	35
	> 40 years	46	12
Position			
	Nurses	198	51
	Doctors	34	9
	Others (Pharmacist etc.)	152	40
Primary work area			
	No specific unit	57	15
	Medicine (non-surgical) unit	93	24
	Surgery unit	45	12
	Obstetrics unit	50	13
	Pediatrics unit	52	13
	Emergency unit	33	9
	Others (i.e. pharmacy etc.)	54	14
Experience			
	< 1 year	133	35
	1–5 years	182	47
	> 5 years	68	18
Work hour (per week)			
	< 24 hours	26	7
	20–39 hours	65	17
	40–50 hours	193	50
	> 50 hours	100	26
Hospital type			
	Public hospital	126	33
	Private hospital	66	17
	Faith-based hospital	192	50

**Dimensions of patient safety culture**: It was found that of the twelve patient safety culture dimensions, two dimensions recorded high positive response rates (≥ 70%). These include teamwork within units (81.5%) and organizational learning (73.1%). Regarding teamwork within units, the areas of strength were respect for each other, support teammates and cooperation. For organizational learning, areas of strength were an evaluation of patient safety interventions and continuity in patient safety improvement. Seven (7) dimensions recorded moderate positive response rates (69% -51%) (i.e. manager’s expectation and teamwork across units).

The dimensions that recorded low positive response rates (≤ 50%) were staffing (34.5%), non-punitive response to error (33.9%) and frequency of events reported (45.7%). For instance, on staffing, more than half (66.4%) of the respondents thought that the staff strength of the hospitals was not enough considering the workload. Details are shown in Table 2. Moreover, the majority (63.8%) of the respondents thought that they worked for more hours than expected. On non-punitive response to error, many of the respondents thought that their mistakes were kept in their personal files. On event reporting, some respondents indicated that they rarely reported adverse events, especially events that did not result in patient harm. (Details are shown in Table 2).

**Table 2:** Frequency distribution on perceptions of patient safety culture among 384 clinical staff in the Upper East region of Ghana, 2017.

**Characteristic**	n	Strongly disagree	Disagree	Neitder	Agree	Strongly agree	% of positive response rate
**Teamwork within units**							**81.5**
People support one another in this unit	384	5.5	3.4	4.1	57.8	29.2	87.0
When one area in this unit gets busy others help	384	11.2	14.3	9.4	43.5	21.6	65.1
When a lot of work needs to be done quickly, we work together as a team to get the work done	384	2.8	3.4	2.9	56.0	34.9	90.9
In this unit, people treat each other with respect	384	3.1	6.8	7.0	54.5	28.6	83.1
**Organizational learning continuous improvement**							**73.1**
We are actively doing things to improve patient safety	384	1.8	6.8	2.3	54.9	34.2	89.1
Mistakes have led to positive changes here	384	12.0	15.8	12.8	47.1	12.3	59.4
After we make changes to improve patient safety, we evaluate their effectiveness	384	4.7	14.6	9.9	54.2	16.6	70.8
**Supervisor expectations & actions promoting patient safety**							**69.7**
My supervisor/ manager says a good word when he/she see the job done according to established patient safety procedures	382	7.6	11.0	5.5	47.4	28.5	75.9
My supervisor seriously considers staff suggestions for improving patient safety	383	9.4	12.8	8.1	46.0	23.7	69.7
Whenever pressure builds up my supervisor/manager wants us to work faster, even if it means taking shortcuts	383	29.0	32.4	15.1	16.7	6.8	61.4
My supervisor overlooks patient safety problems that happen over and over again.	384	41.9	29.7	12.2	6.5	9.7	71.6
**Teamwork across units**							**69.1**
Hospital units do not coordinate well with each other	384	21.1	36.2	10.2	21.1	11.4	57.3
Hospital units work well together to provide the best care for patients	384	3.4	9.6	8.9	52.9	25.2	78.1
It is often unpleasant to work with staff from other hospital units	384	20.8	49.5	11.2	15.4	3.1	70.3
There is good cooperation among hospital units that need to work together	384	5.5	13.7	10.2	53.9	16.7	70.6
**Management support for patient safety**							**60.4**
Hospital management provides a work climate that promotes patient safety	384	9.1	14.1	12.2	49.7	14.9	64.6
The actions of hospital management show that patient safety is a top priority	384	5.6	17.3	10.4	46.1	20.6	66.7
Hospital management seems interested in-patient safety only after an adverse event happens	384	15.5	34.4	9.1	27.1	13.9	50.0
**Handoffs and transitions**							**60.4**
Things fall between the cracks" when transferring patients from one unit to another	383	18.5	34.2	12.5	23.8	11.0	52.7
Important patient care information is often lost during shift changes	384	31.5	35.2	8.6	19.8	4.9	66.7
Problem often occur in the exchange of information across hospital units	384	10.2	40.6	14.6	29.1	5.5	50.8
Shift changes are problematic for patients in this hospital	384	26.3	45.1	9.9	14.3	4.4	71.4
**Overall perception of patient safety**							**53.4**
It is just by chance that more serious mistakes do not happen around	384	21.3	29.2	10.4	29.9	9.1	50.5
Patient safety is never sacrificed to get work done	384	10.2	21.6	5.7	38.5	24.0	62.5
We have patient safety problems in this unit	384	13.8	23.4	8.1	44.0	10.7	37.2
Our procedures and systems are good at preventing errors	384	7.6	16.1	12.8	48.4	15.1	63.5
**Staffing**							**34.5**
We have enough staff to handle the work load	384	31.2	35.2	6.0	21.9	5.7	27.6
Staff in this unit work more hours than is best for patient care	384	9.7	16.9	9.6	43.0	20.8	26.6
We use agency/temporary staff than is best for patient care	384	19.3	31.7	8.3	31.1	9.6	51.0
We work in "crisis mode" trying to do too much, too quickly	384	8.8	24.0	13	36.2	18.0	32.8
**Non-punitive response to error**							**33.9**
The staff feel like their mistakes are held against them	384	11.2	21.6	11.5	38.0	17.7	32.8
When an event is reported, it feels like the person is been written not the problem	384	13.0	26.3	13.5	32.8	14.4	39.3
Staff worry that mistakes they make are kept in their personnel file	384	8.6	21.1	11.2	37.5	21.6	29.7
Characteristic	n	Never	Rarely	Sometimes	Most of the time	Always	% of positive response rate
**Frequency of events reporting**							**45.7**
When a mistake is made but is caught and corrected before affecting the patient, how often is this reported?	384	7.8	21.1	19.3	30.7	21.1	51.8
When a mistake is made but has no potential to harm, how often is this reported	384	11.7	28.9	25.8	18.0	15.6	33.6
When a mistake is made that could harm the patient, but does not, how often is this reported	384	8.8	19.0	20.6	28.1	23.5	51.6
**Communication openness**							**54.4**
Staff will freely speak up if they see something that may negatively affect patient care	384	4.7	10.9	14.1	35.9	34.9	70.3
Staff feel free to question the decisions of those with more authority	384	17.4	24.3	17.4	25.5	15.4	40.9
Staff are afraid to ask questions when something does not seem right	384	28.6	23.5	16.4	19.5	12.0	52.1
**Feedback and communication about error**							**61.3**
We are given feedback about changes put in place based on events reports	384	9.4	14.3	24.5	33.1	18.7	51.8
We are informed about errors that happen in this unit	383	6.6	11.7	14.6	41.3	25.8	67.1
In this unit, we discuss ways to prevent errors from happening again	384	7.3	14.1	13.5	32.8	32.3	65.1

**Patient safety grade**: Regarding the overall perception of patient safety, 7.0% (n = 27) of the respondents perceived patient safety in their units as excellent and 43.8% (n = 168) of the respondents perceived patient safety in their units as very good. About 35% (n = 132) of the respondents perceived patient safety in their units as acceptable, while, 13.8% (n = 53) and 1.0% (n = 4) perceived patient safety in their units as poor and failing respectively.

**Adverse event reporting**: Of the 345 respondents, 73.9% (n = 255) indicated that they had not reported any adverse event over the past twelve months. While, 14.2% (n = 49) of the respondents indicated that they had reported one or two events in the past twelve months, 11.9% (n = 41) indicated that they had reported more than two events in the past twelve months.

### Bivariate analyses

#### Correlation analysis

A positive significant correlation was found between teamwork within units, manager expectation and actions promoting patient safety, organizational learning, management support, feedback and communication about errors, communication openness, teamwork across units, handoffs and transitions, frequency of events reported and non-punitive response to error, and patient safety grade. Details are shown in Table 3.

**Table 3:** Pearson correlation matrix between patient safety culture dimensions and patient safety grade among 384 clinical staff in the Upper East region of Ghana, 2017.

Characteristic	Pearson correlation coefficient	p-value
Teamwork within units	.18	.00
Supervisor expectations and actions promoting patient safety	.15	.00
Organizational learning continuous improvement	.22	.00
Management support for patient safety	.29	.00
Feedback and communication about error	.22	.00
Communication openness	.22	.00
Teamwork across unit	.24	.00
Staffing	-.03	.28
Frequency of events reported	.17	.00
Handoffs and transitions	.13	.01
Nonpunitive response to error	.16	.00
Overall perception of patient safety	.19	.00

### Analysis of variance

One-Way Between-Groups Analysis of Variance was computed to compare mean scores on patient safety grade among the three hospitals. The respondents were categorized into three groups according to hospitals type (Group1: Faith-based; Group 2: Private; Groups 3: Public). There was no statistically significant difference in patient safety grade among the three hospitals: *F* (2, 381) = 2.155, *p* = .117. Concerning working experience, respondents were grouped into three (Group 1: < 1- year of working experience; Group 2: 1 to 5 years of working experience; Group 3: > 5 years of working experience). There was no statistically significant difference in patient safety grade among the three groups: F (2,381) = .021, *p* = .979. To compare working hours on perception of patient safety, respondents were classified into three groups: Group 1: < 40 hours per week; Group 2: 40–59 hours per week; Group 3: ≥ 60 hours per week. The study found no statistically significant difference in patient safety grade among the three groups: F (2,381) = .478, *p* = .620).

In addition, One-Way Between-Group Analysis of Variance was computed to compare mean scores of respondents on event reporting. Respondents were categorized into three groups according to hospital type (Group1: Faith-based; Group 2: Private; Groups 3: Public). There was a statistically significant difference in adverse event reporting among the three hospitals: *F* (2,381) = 4.087, *p* = .02. Despite reaching statistical significance, the actual difference in the mean scores between the groups was quite small. Using the eta squared, the effect size was .02. Post-hoc comparisons using Least Significance Difference (LSD) test indicated that the mean score for Group 1 (*M* = 1.88, *SD* = 1.36) was significantly different from Group 3 (*M* = 1.52, *SD* = .90). Group 2 (*M* = 1.61, *SD* = 1.11) did not differ significantly from either Group 1 or 3. Details are shown in Table 4.

**Table 4:** Multiple comparisons using the Least Significance Difference among 384 clinical staff in the Upper East region of Ghana, 2017.

(I) Name of hospital	(J) Name of hospital	Mean Difference (I-J)	Std. Error	Sig.
Faith-based hospital	Private hospital	.27	.17	.13
	Public hospital	.36	.13	.01
Private hospital	Public hospital	.09	.18	.60
Dependent variable: Number of adverse events reported				

## Discussion

In this study, the overall average positive response rate for the twelve patient safety culture dimensions was 58.1%. This is slightly lower than the AHRQ data (61.0%) [38]. This suggests that healthcare providers in the Upper East region of Ghana feel quite negative towards patient safety culture in their work areas. The study recorded a response rate of 94.5%. Response rates of studies on patient safety culture using the HSOPC questionnaire globally are mixed [39–42]. Some studies report low response rates ranging from 41% to 60% [39, 42, 43]. On the other hand, some studies report relatively high response rates ranging from 91.9% to 97.7% [40, 41, 44]. It appears that response rates on studies on patient safety culture tends to be lower in developed countries such as USA and Japan [39, 43], but rather higher in developing countries such as Saudi Arabia and Brazil [40, 44]. This suggest that patient safety culture is a great source of concern in developing countries, where the prevalence of adverse events tends to be higher [16, 17]. The high response rate in Ghana from this study may be a reflection of this observation. Notwithstanding the preceeding argument, other studies in developing countries report relatively low response rates [45, 46].

The dimension that recorded the highest positive response rate was teamwork within units. This finding is supported by prior studies in Taiwan, Nigeria and Jordan [35, 38, 46]. In a systematic review, Elmontsri, Almashrafi [47] found that teamwork within units was perceived as better than teamwork across units. On the other hand, dimensions such as staffing, non-punitive response to error and frequency of event reported recorded low positive response rates. Previous studies done in Oman, Turkey and Saudi Arabia [9, 10, 46] corroborate these findings. Reporting of adverse events was found to be low. More than half of the respondents indicated that they had not reported any adverse event in the past twelve months to the survey. This may be partly due to the perceived punitive response to error, in view of the fact that more than half of the respondents felt their mistakes were held against them or kept in their personal files. Moreover, more than half of the respondents thought that they were being written up, not the problem. This is not best for positive safety culture and therefore requires urgent attention of the management of the hospitals. Studies have shown that effective response to errors with the main aim of identifying systems errors rather than individual blame is needed to promote a positive patient safety culture [46, 48]. It has been recommended that if medical errors eventually occur, the general system should be looked at, rather than placing blame on the individual who caused the error [49]. A ‘blame-free’ environment where individuals can identify and communicate their mistakes without fear is ideal for good patient care [13, 47]. When medical errors are reported, measures can be implemented to prevent reoccurrence.

There was a significant positive association between patient safety grade and all the patient safety culture dimensions, except staffing. This suggests that an improvement in the dimensions of patient safety culture could lead to an improvement in patient safety. Previous studies report that a positive patient safety culture depends on effective communication, procedure compliance, leadership support, non-punitive response to error, teamwork among others [46, 47]. The study found no statistically significant difference in overall perception of patient safety among the three hospitals. In other words, safety culture in the private hospital was not perceived to be better or worse than the safety culture in the faith-based and public hospitals. This is consistent with Ejajo, Arega, and Batebo [37], who found no significant difference in the overall perception of patient safety among three hospitals in Ethiopia.

Regarding event reporting, a significant difference was found among the three hospitals. The faith-based hospital reported more events than the public hospital. One of the possible reasons underlying this finding is the differences in capacity. The faith-based hospital is the largest health facility in the Upper East region. It is therefore expected that the faith-based hospital would have a more advanced reporting system than the other hospitals.

### Implications for practice and policy

The findings of the study have implications for health policy and practice. To improve event reporting, the management of the hospitals would have to implement strategies to promote a ‘blame-free’ environment where clinical staff can confidently report medical errors or mistakes without fear of being held accountable for their mistakes. Healthcare providers are more likely to report adverse events if they are assured that their mistakes would not be held against them. This study provides valuable information for patient safety improvement and interventions, however, it is not devoid of limitations. The Upper East region is one of the deprived regions in Ghana, in terms of health care resources. Therefore, a generalization of the findings must be done with caution. In this regard, the findings of this study may differ from the findings of studies done in less deprived regions. It is therefore suggested that future studies consider including hospitals in less deprived regions. Notwithstanding, this is one of the few studies in Ghana that compares patient safety culture among public, private and faith-based hospitals.

## Conclusion

The findings of this study reveal healthcare providers’ perceptions of patient safety culture in three hospitals. The overall perception of patient safety culture did not differ significantly among the hospitals. Teamwork within units was perceived to be high, whilst non-punitive response to error was perceived to low. Reporting of adverse events was low, coupled with perceived punitive response to errors. Going forward, healthcare policy-makers and managers should consider patient safety culture a top priority, and also create a blame-free environment that promotes event reporting.
